# Functional responses of *Daphnia magna* to zero-mean flow turbulence

**DOI:** 10.1038/s41598-019-40777-2

**Published:** 2019-03-07

**Authors:** Teresa Serra, Mara F. Müller, Jordi Colomer

**Affiliations:** Department of Physics, University of Girona, 17003 Girona, Spain

## Abstract

*Daphnia* are important to understanding the biogeochemistry of aquatic ecosystems, mainly because of their ability to filter bacteria, algae and inorganic particles as well. Although there are many studies on the general effects that biotic and abiotic stressors, increased temperature and hypoxia, salinity, metals, pharmaceuticals, pesticides, etc., have on *Daphnia* populations, little is known about the impact elevated turbulence has. Here, we show that turbulence affects *Daphnia magna* survival, swimming behaviour and filtering capacity. Our data demonstrate that altering their habitat by induced mixing from turbulence, induces an increased filtering capacity of the *Daphnia magna* individuals, provided the level of background turbulence (defined by the dissipation of turbulent kinetic energy) is lower than ε = 0.04 cm^2^ s^−3^. The filtering capacity reduced exponentially with increasing ε, and at ε > 1 cm^2^ s^−3^ both mobility and filtration were suppressed and eventually led to the death of all the *Daphnia magna* individuals.

## Introduction

*Daphnia* (a genus from the order of Cladocera) are known to be ubiquitous in many aquatic ecosystems. They are filter feeders that not only feed on small bacteria and phytoplankton^1^, but also on small wastewater sludge particles^2–4^. The *Daphnia* genus serve as food for fish and invertebrates and are key organisms in terms of the water quality in lakes and ponds. Filter feeding is common in aquatic plankton organisms that ingest small suspended particles which are then removed from the water column^5^. Particle interception by most zooplankton is regulated by the encounter rates between the suspended particles, i.e., prey, and the filter feeders, i.e., predators. The transition from encountering prey to successfully capturing it depends on the capture efficiency coefficient α that represents the successful encounters between prey and predator^5–7^. Turbulence increases the encounter rate between prey and predators. This is extremely important for low mobility predators and rather important for cruising predators as well^8^. While turbulence is also of vital importance for meso-sized (mm to cm) predators, it is less so for smaller and larger predators^8^.

*Daphnia* is a keystone genus that is widespread in most freshwater ecosystems, and is routinely exposed to a multitude of anthropogenic and natural stressors^9^. Such stress produces disturbances to *Daphnia* populations because, as they cannot keep up with the changes, this results in a decline in the number of individuals^10^. Swimming behaviour^11^, survival^12^, growth and reproduction^13^ are, among others, the key characteristics of *Daphnia* that determine to what degree biotic effects such as metals^14^, pharmaceuticals^15^ and pesticides will impact on them. Besides biotic parameters, *Daphnia* are intolerant of and stressed by abiotic conditions such as temperature^14,16–18^, hypoxia, salinity^19^ and hydrodynamics^4^ which have been found to alter their behavior. So far, no study has documented the effects zero-mean flow turbulence has on *D. magna* individuals.

In freshwater ecosystems the so-called pusher swimmers^20,21^ like the freshwater *D. magna* individuals, produce mean dissipation rates from 0.034 to 0.018 cm^2^ s^−3^, which are associated to viscous trail dissipation^22^, although when forming schools *Daphnia* might produce maximum dissipations of ε = 2.8 cm^2^ s^−3 ^^21^. Therefore, like many zooplankton species, *D. magna* might potentially be important for vertical mixing in weak mixing ambient flows^21,23^. In contrast, in aquatic ecosystems, turbulence plays an additional role as a stressor by modifying the swimming capacity and survival of organisms^4^, to shifting community structure^24^ or producing active cyclomorphosis^25^. The ingestion rate of weak planktonic protozoa swimmers rises sigmoidally with increasing turbulence that is characterised by shears of 1–10 s^−1^ which correspond to turbulent kinetic energy dissipations in the order of 1 to 0.01 cm^2^ s^−3 ^^26^. That said, at turbulence characterized by shears greater than 1 cm^2^ s^−3^, feeding was suppressed^26^.

In this work, the effect turbulence intensity has on the swimming behaviour, filtering capacity and survival of *D. magna* is studied. We exposed *D. magna* individuals to zero-mean flow turbulent conditions, which were generated by an oscillating grid, and compared them to those under steady flow control conditions. The modification of the swimming behaviour, survival rate and food filtration of *D. magna* in a turbulent domain characterised by shears of 0.55–9.80 s^−1^ corresponding to turbulent kinetic energy dissipations ranging from 0.003 to 0.932 cm^2^ s^−3^ is presented. Oscillating grid devices have been previously used to test the behaviour of zooplanktonic populations under turbulent regimes^27^. The turbulence generated by the oscillating grid in the present study ranges from 0.003 cm^2^ s^−3^ to 1.4 cm^2^ s^−3^. This range of turbulence is characteristic of mean turbulence intensities in shallow littoral zones in lakes and ponds, with mean values from 0.001 cm^2^ s^−3^ to 2 cm^2^ s^−3 ^^24,28,29^. The littoral zones of lakes and ponds are regions with limited advection and the main source of turbulence is due to the action of the wind at the surface, or due to night convection, that decays with distance to the source. For this reason, the vertical decay of the turbulent kinetic energy produced by an oscillating grid has been found suitable for this study. *D. magna* are organisms ubiquitously found in lakes and ponds. Therefore, it is expected that this is the turbulence that *D. magna* will encounter in natural systems.

## Material and Methods

### The oscillating grid system

**Figure 1:**
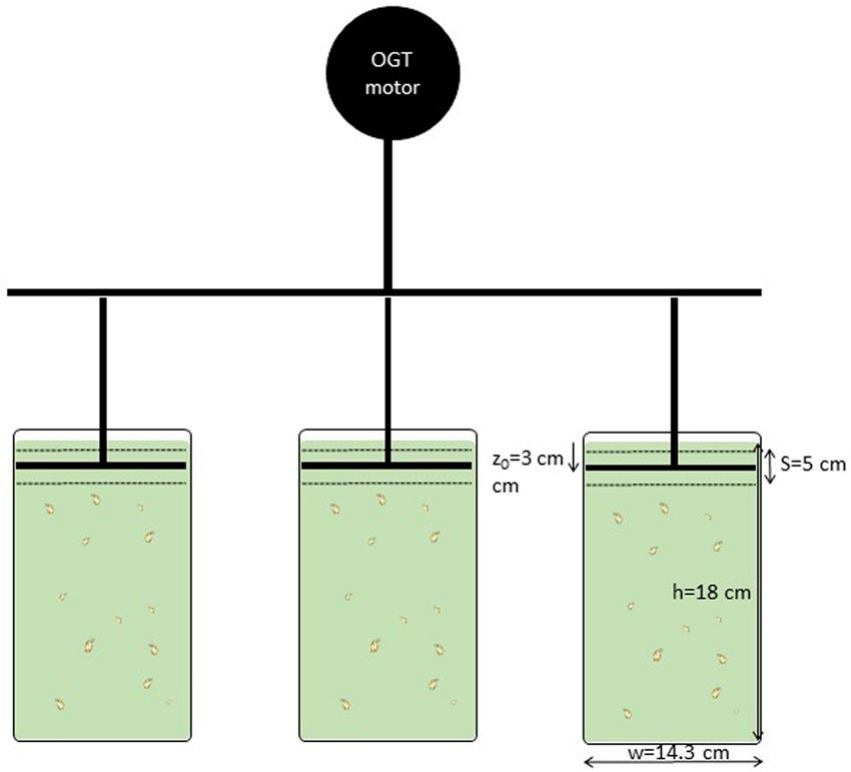
Schematic view of the set-up used for the experiments.

An oscillating grid system was used in this study (Fig. 1). The device was similar to that described in Peters and Gross^30^ and Colomer *et al*.^31^. Movement was provided by a controlled motor attached to a frame holding three grid shafts (Fig. 1). Thus, three replicate containers could be used simultaneously. Each grid oscillated inside a Plexiglas container with a working height of 0.18 m and a diameter of 0.143 m. Therefore, the working volume of each container was 2.89L.

Grids oscillated vertically with frequencies ranging from 0.25 to 2 Hz (Table 1). The oscillating amplitude (stroke) was 5 cm. Grids were made of cylindrical stainless steel, with a diameter of 4.0 mm and a mesh size of 1.5 cm. Solidity, that is, the percentage of solid surface perpendicular to the direction of movement, was 40.2%; similar to the 37.8% used by Guadayol *et al*.^32^. With this set-up, the oscillating grids were always switched on and the grid situated in its mid position: 3.0 cm from the top of the working height cylinder and defined here as the virtual origin, z_0_. The rectilinear oscillating motion of the grid was ensured by constraining the movement of the connecting grid shafts so that they travelled along a guide rail using precision bearings^33^. In addition, to avoid secondary circulation in the tank^4^ the grid was designed by deeming that the distance between the end of the grid and the wall of the cylinder was equal to half a square of the grid.

**Table 1:** Hydrodynamic conditions tested with the oscillating grid device. f is the oscillating frequency of the grid, ε is the mean energy dissipation averaged over the vertical working depth from equation (6), G is the mean shear rate averaged over the working depth and λ is the mean Kolmogorov microscale for each hydrodynamic condition following equation (10).

f(Hz)	ε(cm^2^ s^−3^)	G(s^−1^)	λ(mm)
0	0	0	0
0.3	0.003	0.55	1.33
0.4	0.007	0.85	1.07
0.5	0.012	1.13	0.93
0.6	0.020	1.43	0.83
0.7	0.044	2.11	0.68
0.9	0.073	2.71	0.60
1.0	0.112	3.35	0.54
1.1	0.161	4.02	0.49
1.3	0.219	4.69	0.46
1.4	0.294	5.42	0.42
1.5	0.397	6.30	0.39
1.6	0.500	7.07	0.37
1.8	0.620	7.88	0.35
1.9	0.792	8.90	0.33
2.0	0.953	9.76	0.32
2.3	1.40	10.61	0.30

### Fundamental theory of grid turbulence

The turbulence produced by the oscillating grid is characterized by its zero-mean flow and is two-dimensionally homogeneous in a certain region away from the grid^34–36^. The horizontal and vertical root-mean square turbulence velocities (u′ and w′) decay linearly with depth, while the integral length scale increases with distance z from the grid in the form:

1 $$u^{\prime} =v^{\prime} ={C}_{1}{M}^{0.5}{S}^{1.5}f{z}^{-1}$$

2 $$w^{\prime} ={C}_{2}{M}^{0.5}{S}^{1.5}f{z}^{-1}$$

3 $$l={C}_{3}z$$

where u′, v′, and w′ are the RMS velocities in the three directions (x, y, z), respectively; M is the mesh size (defined as the distance between the centers of two grid bars); C_1_ = 0.22, C_2_ = 0.25 and C_3_ = 0.10 are constants, depending on the grid geometry^33^. The turbulent kinetic energy can then be expressed as:

4 $$k=\frac{1}{2}(2{C}_{1}^{2}+{C}_{2}^{2}){({M}^{0.5}{S}^{1.5}f{z}^{-1})}^{2}$$

In the oscillating grid devices, the fundamental theory focuses either on the vertical distribution of turbulent kinetic energy^37^ or on the mean turbulent kinetic energy dissipation rate, ε^31,38,39^,

5 $$\varepsilon =\gamma u{^{\prime} }^{3}{l}^{-1}$$

where γ is a constant taken as equal to 0.8, l is the integral length scale using the distance from the grid, and u′ is the mean velocity integrated over the whole working depth. The dissipation rate ε is related to the mean shear rate G as

6 $$G={(\varepsilon /\nu )}^{1/2}$$

The parameter G has been widely used to describe the aggregation of particles by oscillating grid devices under high turbulence^40^ and also in low-shear flows^31^.

Using equations (1), (5) and (6), it is possible to obtain the dependence of the shear rate on grid characteristics, the frequency of the grid and the depth according to

7 $$G=\sqrt{\frac{\gamma }{\nu {C}_{3}}{C}_{2}^{3}\frac{{M}^{3/2}{S}^{9/2}{f}^{3}}{{z}^{4}}}$$

which can be reduced to

8 $$G=0.01811{f}^{3/2}{z}^{-2}$$

when considering a mesh size of 1.5 cm.

The mean shear rate in the containers can be calculated by taking the average of G, and integrating G over the depth from z = z_o_ to the working height. For the frequencies studied and the working height considered, G was found to be within the range of 0.55 to 9.76 s^−1^ (Table 1), therefore in the range of low shear flow^31^. The Reynolds number of the grid (Re_G_ = u′d/ν) can be calculated as follows:

9 $${{\rm{Re}}}_{G}=\frac{{C}_{1}{C}_{3}{M}^{0.5}{S}^{1.5}f}{\nu }$$

after considering equations (1) and (3), Re_G_ is independent of the distance from the grid. Re_G_ ranged from 9 to 63, depending on the value of f, and in all cases corresponded to the turbulent regime. Finally, the size of the smallest turbulent eddies within the fluid was estimated from the Kolmogorov length scale λ, according to:

10 $$\lambda ={({\nu }^{3}/\varepsilon )}^{1/4}={(\nu /G)}^{1/2}$$

The Kolmogorov length scale was found to vary between 1.33 to 0.32 mm (Table 1). Therefore, for the smallest values of the shear rate, the Kolmogorov scales were found to be of the same order as the size of the *Daphnia magna* individuals.

### *D. magna* characteristics

*D. magna* individuals were taken from two *D. magna* cultures which had been maintained at a constant temperature (20.0 ± 0.3 °C) and daylight photoperiod for 2 years in two 40 L containers in the facilities at the University of Girona. The containers are constantly supplied with air to ensure water oxygenation. We chose mineral water rich in calcium (constant value of 35.7 mg/L) to avoid calcium depletion which can reduce *D. magna* body size^41^. The *D. magna* culture was fed every four days on a combination of Baker’s yeast (*Saccharomyces cerevisiae*) and spirulina powder using a ratio of 25 to 75%, respectively. Fifty per cent of the water content in the cultures was renewed each week.

### *Control* and *D. magna* experiments

Sixteen control experiments were carried out without *D. magna* individuals. The oscillating grids were switched on in each of the three system replicates filled with 2.89 L of bottled mineral water and 30 ml of spirulina suspension. The spirulina suspension was prepared as follows: 1 g of spirulina powder diluted in 1 l of mineral water, mixed for 60 s at 100 rpm and left for 1 h so that large spirulina particles settled at the bottom. A supernatant was used as the spirulina suspension for the experiments. All the experiments related to *Daphnia* filtration lasted 4 h, following Pau *et al*.^2^ who showed that the decrease in the particle concentration resulting from the ingestion of *D. magna* individuals decreases exponentially with time. Pau *et al*.^2^ choose the characteristic time in all the experiments to be time t when the initial concentration decreased in e^−1^ = 0.37 i.e., approximately 4 hours.

Zero-mean turbulence experiments began at controlled shear rates of 0 s^−1^ and between 0.55 and 9.76 s^−1^ (Table 1). At a shear rate of 0 s^−1^ the experiment was fully dominated by the sedimentation of the spirulina particles, while the experiments carried out with shears between 0.55 and 9.76 s^−1^ were dominated by both the sedimentation of spirulina particles and the effect the induced mixing by the grids had in the containers. For the experiments without *D. magna*, the no-shear experiment proved that after 4 hours of experiments c/c_o_ was 0.54, indicating that sedimentation contributed to 46% of the particle removal. For experiments with shear, c/c_o_ ranged from 0.58 at ε = 0.003 cm^2^ s^−3^ to 0.61 at ε = 0.953 cm^2^ s^−3^, indicating that sedimentation was reduced within the range of 42% to 39% for the range of dissipations studied.

The spirulina particle size distribution in the suspension was measured with a laser particle size analyser Lisst-100× (Sequoia Inc.). Samples from each replicate were taken at 0 hours and 4 hours and analysed to determine suspended particle concentration. The laser analyser consists of a laser beam and an array of detector rings of progressive diameters that allow the light received at the scattering angles of the beam to be analysed. The device measures the particle volume concentration of particles for 32 size-classes, (logarithmically distributed in the size range of 2.5–500 μm), using a procedure based on the diffraction theory of light. The analyser has been found to perform well in determining particle size distribution and concentration for both organic^42^ and inorganic particles^43,44^ in water suspension. Particle concentration was calculated by integrating the concentration of the particles within the *D. magna* feeding range i.e., from particles of 2.5 to 30 μm in diameter^45^. Therefore, the volume concentration of particles within the *D. magna* feeding range of 2.5 to 30 μm was used as a proxy to evaluate particle removal.

Each experiment with the *D. magna* population began by introducing a concentration of 50 *D. magna* individuals l^−1^ (hereafter ind l^−1^) in each of the three system replicates filled with 2.89 l of bottled mineral water and 30 ml of spirulina suspension. For each experiment carried out with *D. magna*, the individuals were collected from the cultures held in the facilities at the University of Girona.

A total of forty-eight experiments were carried out with *D. magna* individuals and were distributed depending on the size of the *D. magna* individuals. Sixteen experiments were done with *D. magna* individuals presenting mean sizes of L_1_ = 1.25 ± 0.3 mm, 16 experiments with *D. magna* individuals presenting mean sizes of L_2_ = 1.50 ± 0.3 m, and 16 experiments with *D. magna* individuals presenting mean sizes of L_3_ = 1.85 ± 0.5 mm. *D. magna* individuals were collected from the cultures with appropriate mesh spacings in order to retain individuals of different sizes (L_1_, L_2_ and L_3_).

All the tests, protocols and analysis with *D. magna* were carried out aligning with the international ‘OECD/OCDE Guidelines for the Testing’^46^ and the ‘Protocol of sampling and laboratory tests of invertebrates’ code ML-L-I-2013^47^ of the Ministerio de Agricultura, Alimentación y Medio Ambiente of the Spanish Government.

### *D. magna* trails and speed

After 4 hours of exposure to the experimental conditions considered in each case, a video recording of the trajectories of 25 *D. magna* was used to obtain information on their swimming velocity. The swimming patterns of the *D. magna* individuals were characterized by cruising, hopping and sinking, and looping^48^ and were differentiated based on the net to gross displacement ratio^22^. The three patterns observed were cruising, in which organisms swam in a near-straight trajectory; hopping and sinking, in which organisms move in a succession of ascending and descending pathways; and looping, in which organisms moved following circular or spiral-like pathways^22^. In this range, active propulsion and inertial or gravitational movement is fully three-dimensional with no preferential direction. However, despite having a wide variety of *D. magna* movements, we chose cruising as the one to be considered in the present study because it was the most frequent movement observed. Analysis of the *D. magna* velocity was carried out by videotaping the movement of the individuals. The lengths of the trajectories considered for the analysis were between 6 and 7 cm. When *D. magna* entered in the region of the oscillating grid region they were lost for a time lag and therefore were discarded thereafter. The camera recorded 25 frames per second and the *D. magna* trails were recorded for 1 min for each case, giving a total of 1500 frames. These frames were analysed with ImageJ software using the mTrack plug-in^4,12,49^. The mean size of the *D. magna* individuals was also obtained from the ImageJ software video recording of the trajectories made by 25 individuals during each set of experiments.

### *D. magna* filtration capacity

Since the temporal evolution of the suspended particle concentration decreased exponentially, concentration can be described by an exponential decay equation as follows (Pau *et al*.^2^):

11 $$c={c}_{0}{e}^{-{\rm{k}}{\rm{t}}}$$

where k is the total rate of particle removal by both sedimentation (k_s_) and *D. magna* filtration (k_Dph_), i.e. k = k_s_ + k_Dph_. From Eq. (11) k can be solved following:

12 $${\rm{k}}=-\,\,1/{\rm{t}}\,\mathrm{ln}({\rm{c}}/{{\rm{c}}}_{0})$$

and k_s_ can be determined from those experiments without individuals of *D. magna* (in which k_Dph_ = 0). Therefore, k_Dph_ will be calculated for the rest of the experiments. The rate of decrease due to *D. magna* filtration is a function of the filtering rate of each *D. magna* individual (F, in ml ind^−1^ l^−1^) and the *D. magna* concentration in such a way that

13 $${{\rm{k}}}_{{\rm{Dph}}}={\rm{F}}\times {{\rm{C}}}_{{\rm{Dph}}}$$

The kinetics of particle-particle collision in a system of two populations in a sheared fluid can be calculated as a function of the shear rate G. In such a case, the collision frequency function β can be determined from the shear rate and the size of the colliding particles following (Lick and Lick, 1988):

14 $$\beta =4.2\pi G{(L+{d}_{p})}^{3}$$

where d_p_ is the diameter of the food particle colliding with *D. magna*. Although this function has been demonstrated to determine the filtration capacity of *D. magna* of a certain size^4^, this equation applies to *D. magna* individuals of sizes L below the Kolmogorov length scale λ^8^. For *Daphnia* lengths L larger than the Kolmogorov length scale the collision frequency function between particles can be related to the dissipation and the *D. magna* size through^8^

15 $$\beta ={\varepsilon }^{1/3}{(L+{d}_{p})}^{7/3}$$

If we assume that, in general, the mean Kolmogorov length scale λ is in the order of the *Daphnia* size or larger in some of the cases, to better describe the removal of suspended particles by *D. magna* equation (15) should be used instead of (14). Therefore, the rate of small particles captured by a *D. magna* individual on a particle suspension of concentration c can be written in the following form^4^

16 $${R}_{Dph}=\alpha \beta c+{R}_{Dph}(0)$$

where α is the capture efficiency for each *D. magna* individual, G is the shear rate, and R_Dph_(0) = K_Dph_(0) × c is the particle removal rate by the *D. magna* individual in a steady flow (i.e. at G = 0 s^−1^). Therefore, the rate of the decrease in small suspended particles due to *D. magna* feeding can be written as

17 $$\frac{1}{{C}_{Dph}}\frac{dc}{dt}=-\,\alpha \beta c-\frac{{k}_{Dph}(0)c}{{C}_{Dph}}$$

and by merging equation (11) and equation (17) we obtain

18 $$-\frac{{c}_{0}}{{C}_{Dph}}{k}_{Dph}(G){e}^{-{k}_{Dph}(G)t}=-\,\alpha \beta c-\frac{{k}_{Dph}(0)c}{{C}_{Dph}}$$

and with equation (11), equation (18) can be written as

19 $$\frac{{k}_{Dph}(G)}{{C}_{Dph}}=\alpha \beta +\frac{{k}_{Dph}(0)}{{C}_{Dph}}$$

and therefore

20 $${k}_{Dph}(G)=\alpha \beta {C}_{Dph}+{k}_{Dph}(0)$$

using equations (13), (15) and (20), the filtration results in a function of G, α and L that can be written as

21 $$F=\alpha {\varepsilon }^{1/3}{L}^{7/3}+F(0)$$

where F(0) = k_Dph_(0)/C_Dph_, and where we have assumed that since ${{\rm{d}}}_{{\rm{p}}}\ll {\rm{L}},\,{\rm{L}}+{{\rm{d}}}_{{\rm{p}}}\cong {\rm{L}}$.

## Results

**Figure 2:**
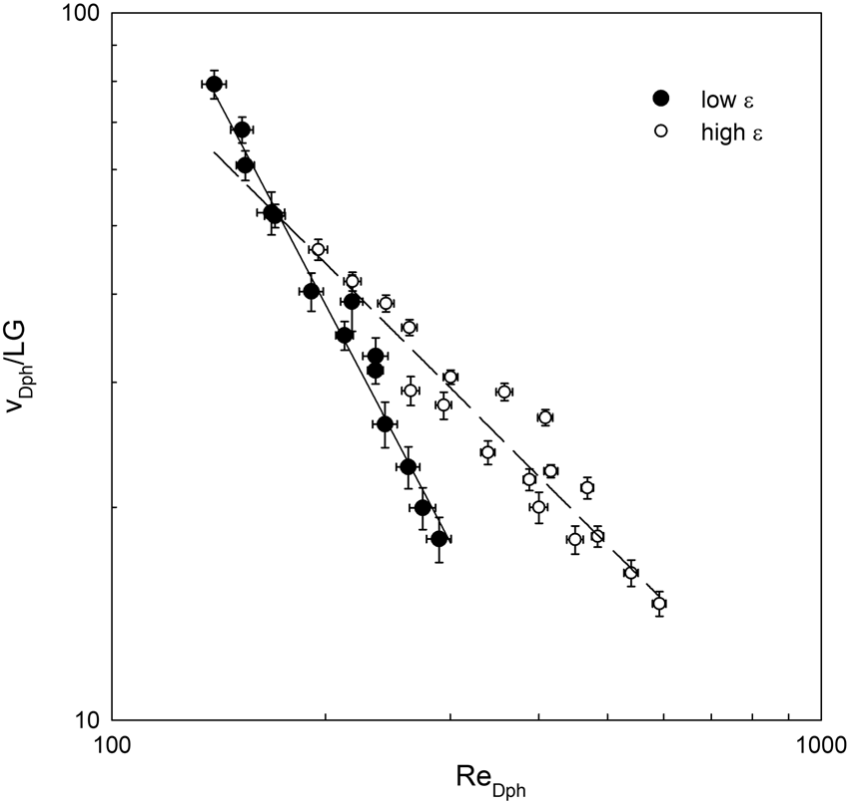
Non-dimensional *D. magna* velocity (v_Dph_/LG) versus the *D. magna* Reynolds number (Re_Dph_ = v_Dph_L/ν). Lines represent the best fit to the experimental results for the two ranges of ε considered. For low ε, the best-fit equation was v_Dph_/LG = 960930Re_Dph_^−1.91^ (R^2^ = 0.9732, 99% of significance). For high ε, the best-fit equation was v_Dph_/LG = 8446.6Re_Dph_^−1^ (R^2^ = 0.9186, 99% of confidence). Vertical and horizontal error bars represent the propagation of uncertainty of the ratio v_Dph_/LG for the vertical and for Re_Dph_ = v_Dph_L/ν for the horizontal. The standard deviation of the different measurements taken was considered as the uncertainties in the measured variables.

The non-dimensional velocity of *D. magna* (v_Dph_/LG) was plotted versus the *D. magna* Reynolds number (Re_Dph_) for all the experiments carried out (Fig. 2). A power dependence was found between v_Dph_/LG and Re_DPh_. Different power trends were found depending on the level of ε. The change from low ε to high ε also depended on the size of the *D. magna*. For large *D. magna* of 1.85 mm, the critical ε was at ε_cr_ = 0.16 cm^2^ s^−3^, for *D. magna* of 1.50 mm, the critical ε was at ε_cr_ = 0.11 cm^2^ s^−3^ and for *D. magna* of 1.25 mm the critical ε was ε_cr_ = 0.08 cm^2^ s^−3^. For low ε < ε_cr_, v_Dph_/LG decreased markedly with Re_Dph_ with a dependence v_Dph_/LG = 960930Re_DPh_^−1.91^ (R^2^ = 0.9732 and with 99% significance). This results in an equation for v_Dph_ = 113.74ν^0.66^L^−0.31^G^0.34^ that, by using equation (6), can be written as

22 $${v}_{Dph}=113.74{\nu }^{0.49}{L}^{-0.31}{\varepsilon }^{0.17}$$

Therefore, for ε < ε_cr_, results indicate that v_Dph_ depended on both L and ε.

For dissipation rates ε > ε_cr_, v_Dph_/LG decreased with Re_Dph_ as v_Dph_/LG = 8446.6Re_Dph_^−1^ (R^2^ = 0.9186 and 99% confidence). This results in an equation for v_Dph_ = 94.06(νG)^1/2^ that, by using equation (6), can be written as

23 $${v}_{Dph}=94.06{\nu }^{0.25}{\varepsilon }^{0.25}$$

Therefore, for ε > ε_cr_, results indicate that v_Dph_ depended only on ε and not on L, contrary to what was found for ε < ε_cr_.

**Figure 3:**
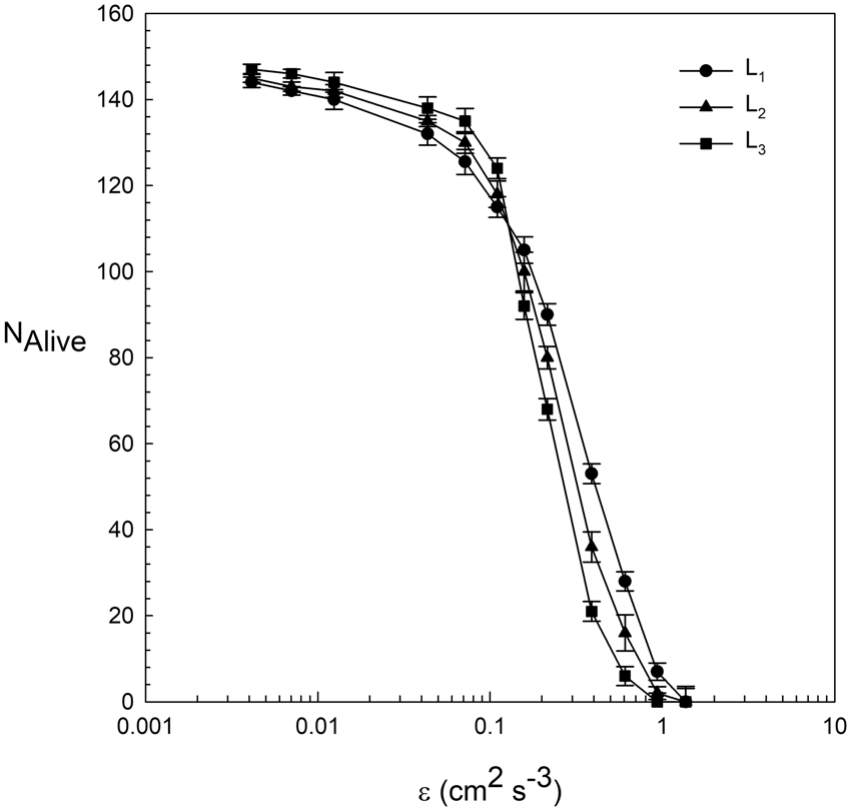
Number of *D. magna* alive versus the dissipation ε for the three *D. magna* of different lengths (L_1_ = 1.25 mm, L_2_ = 1.50 mm and L_3_ = 1.85 mm) studied. Vertical error bars represent the standard deviation over the different replicates made. ANOVA analysis showed that significant differences occurred in the number of *D. magna* alive (N_alive_) between *D. magna* sizes (L_1_, L_2_, L_3_) at the same dissipation rates, ε (P < 0.05).

The number of *D. magna* alive after 24 h was plotted versus the energy dissipation ε (Fig. 3). For ε below 0.16 cm^2^ s^−3^, the number of *D. magna* decreased with ε, with numbers of *D. magna* alive above 120 after 24 h. For ε > 0.16 cm^2^ s^−3^, the number of *D. magna* alive decreased sharply with ε. The largest daphnia showed the greatest decrease with ε > 0.16 cm^2^ s^−3^. It is interesting to note that for ε < 0.16 cm^2^ s^−3^, the number of large *D. magna* of L_3_ was slightly above that for small *D. magna* of L_1_, when considering the error marge of these data points. However, for ε > 0.16 cm^2^ s^−3^, the number of large *D. magna* of L_3_ decreased faster with ε than for small *D. magna* of L_1_ (Fig. 3). No *D. magna* remained alive for ε > 1 cm^−2^ s^3^.

**Figure 4:**
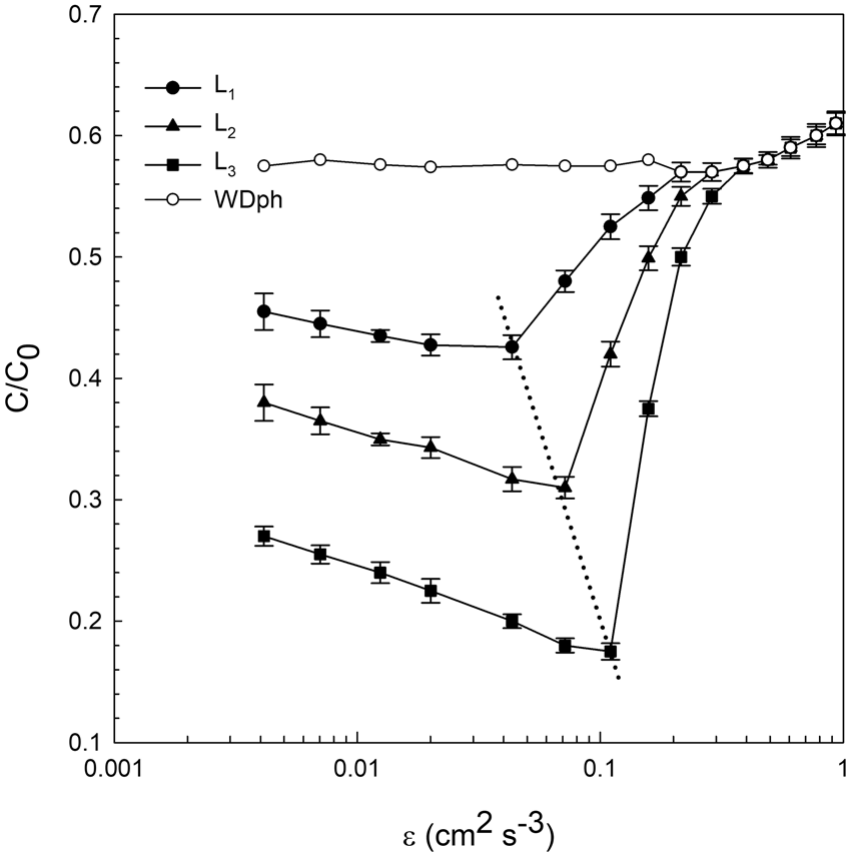
Ratio c/c_0_ versus ε for the experiments with the three *D. magna* sizes (L_1_ = 1.25 mm, L_2_ = 1.50 mm and L_3_ = 1.85 mm) and for the experiments without *D. magna*. The dashed line represents the shift of the minimum of c/c_0_ as L increases. Vertical error bars represent the vertical uncertainty. For this estimation, the uncertainties in each c and c_0_ were considered as the standard deviation of the results from the different replicates made.

The ratio (c/c_0_) was plotted versus ε for all the experiments carried out (i.e. with and without *D. magna*, see Fig. 4). For the experiments without *D. magna*, the ratio c/c_0_ was constant for ε < 0.3 cm^2^ s^−3^. For ε > 0.3 cm^2^ s^−3^, c/c_0_ increased with ε. For experiments with *D. magna* and for ε < 0.3 cm^2^ s^−3^, c/c_0_ was below that for experiments without *D. magna*. The largest *D. magna* individuals had the lowest c/c_0_. Furthermore, c/c_0_ for experiments with *Daphnia* decreased as ε increased, provided that ε remained below ε = 0.04, 0.08 and 0.1 cm^2^ s^−3^ for the *D. magna* lengths L_1_, L_2_ and L_3_. Above this ε, c/c_0_ increased markedly and at ε = 0.3 cm^2^ s^−3^, c/c_0_ was the same for all *D. magna* sizes and also equal to the case without *D. magna*.

**Figure 5:**
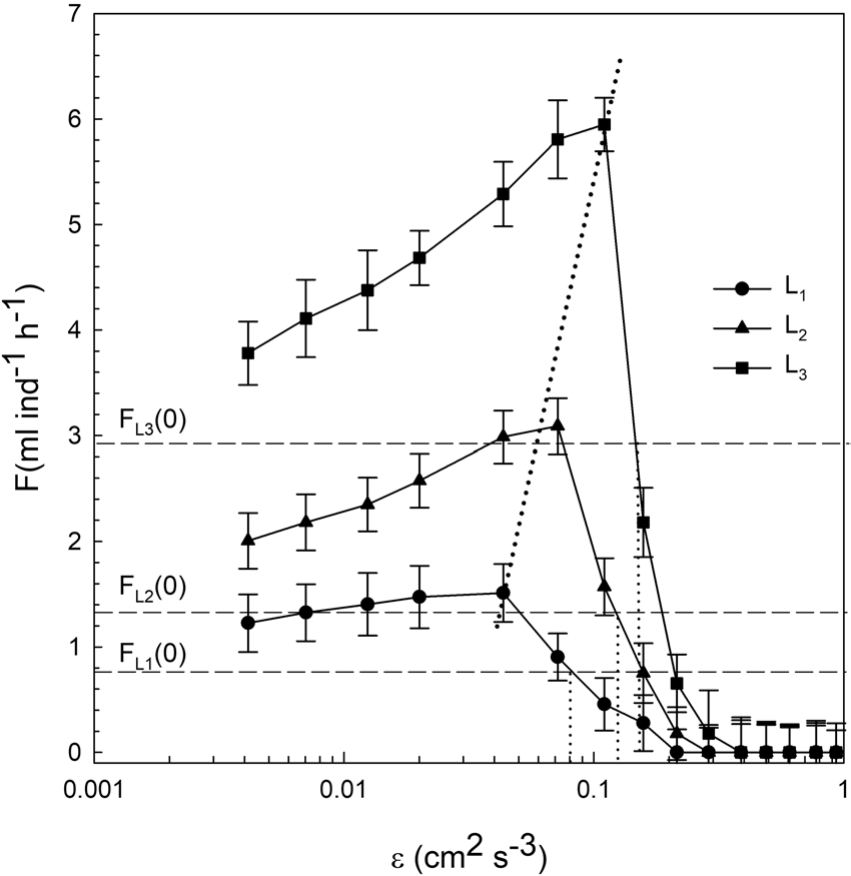
*D. magna* filtration F versus ε for the experiments carried out with the *D. magna* of the three different lengths (L_1_ = 1.25 mm, L_2_ = 1.50 mm and L_3_ = 1.85 mm). The horizontal dashed lines represent the *D. magna* filtration at ε = 0 cm^2^ s^−3^ for the three different *D. magna* lengths studied. The sloping dashed line represents the shift to greater ε of the maximum filtration with increasing ε. Vertical error bars have been estimated from the uncertainty propagation knowing the uncertainty in c/c_0_.

*D. magna* filtration was calculated by using equations (12) and (13) for each value of c/c_0_. Two different regions were observed for F depending on dissipation ε (Fig. 5). For ε < ε_cr_, the filtration rate increased with ε by ∼2 times that found for ε = 0 cm^2^ s^−3^ for all *D. magna* lengths (Fig. 5). For ε > ε_cr_, the filtration decreased markedly with ε, with filtrations below F(0) for ε_cr_ > 0.08 cm^2^ s^−3^, ε_cr_ > 0.11 cm^2^ s^−3^ and ε_cr_ > 0.16 cm^2^ s^−3^ for *D. magna* of lengths L_1_, L_2_ and L_3_, respectively. For ε = 0.3 cm^2^ s^−3^, the filtration was close to zero for all the *D. magna* lengths considered.

**Figure 6:**
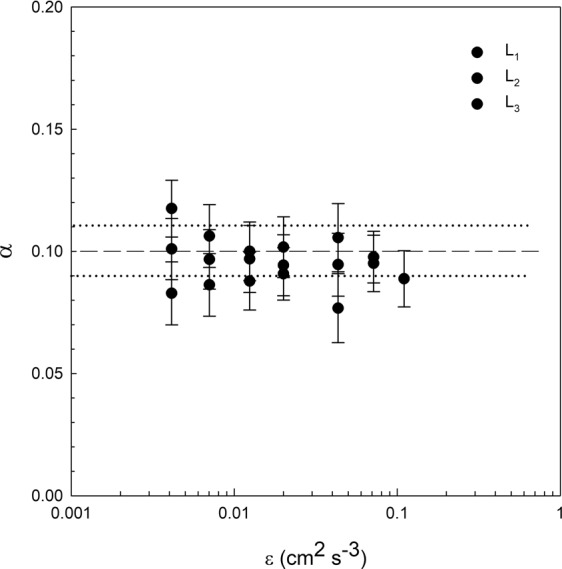
Capture efficiency coefficient α versus ε for the different *D. magna* lengths considered (L_1_ = 1.25 mm, L_2_ = 1.50 mm and L_3_ = 1.85 mm). The solid horizontal line represents the mean value of the different α obtained. The dashed horizontal lines represent the standard deviation of the points around the mean value. Vertical error bars have been estimated from the propagation of the uncertainty in the filtration.

From equation (21), the value of the capture efficiency was calculated. In Fig. 6 α = (F-F(0))/(ε^1/3^L^7/3^) was plotted versus ε. α was constant with ε, with a mean value of 0.10 ± 0.01. This finding indicates that the model considered here in equation (21) is able to predict the filtration rate dependence of *D. magna* using both the *D. magna* length scale L and the dissipation of the fluid ε.

## Discussion

The swimming velocity of *D. magna* in a quiescent flow increased with *its* body length. This is in accordance with the findings of other authors^22^. However, under turbulence *D. magna* swimming speeds changed and their behaviour depended on the level of turbulence which is characterized by the dissipation of turbulent kinetic energy. For dissipation rates below a critical level that depended on the length of the *D. magna*, the *D. magna* swimming velocity depended on both the *D. magna* length scale and the dissipation of turbulent kinetic energy. Therefore, *D. magna* still had some control over their movements through a weak dependence of v_Dph_ on L added to the dependence of v_Dph_ on ε. It is interesting to notice that the dependence of v_Dph_ with L was inverse, i.e., L increases when v_Dph_ decreases indicating that under the same ε small *D. magna* will have greater velocities than large *D. magna*, i.e., small *D. magna* will follow the flow dynamics better. In this region of ε < ε_cr_, the *D. magna* velocity increases with ε, indicating that the greater the ε, the greater the *D. magna* velocity. In turbulent flows with ε > ε_cr_, the velocity of *D. magna* followed a power dependence that only depended on ε and was independent of the length of the *D. magna* individuals. This indicates that in this regime the *D. magna* movement was completely dominated by the flow. Thus, the allometric equation found for *D. magna* swimming velocity in quiescent flows^22^ would not apply when predicting their swimming velocity in turbulent environments. Therefore, since turbulence is ubiquitous in all aquatic systems, this must be considered when assessing the movement of such organisms in the water column. In the highest turbulent flows, a greater number of *D. magna* hoppings were observed compared to that in quiescent flows. These hoppings were mainly restricted to the bottom layer of the beaker. *D. magna* situated above this layer were transported by the flow.

Moderate turbulence levels have been found to increase the clearance rate of moderate and weak swimmers in many lakes and ponds^8,26^. However, high turbulence supressed the clearance rate to below that in still water^26^. Larger *D. magna* individuals (1.85 mm) may present filtrations four times those of 1.25 mm individuals, indicating that filtration is not only a function of shear, but also a power function of their size (L^7/3^). Moderate dissipation rates enhance the filtration rate, whereas high levels of turbulence inhibit *D. magna* filtration to values below those in quiescent flows. Therefore, turbulence could be an important factor in controlling both the bacterial and the phytoplankton populations in aquatic systems through direct positive feedback on *D. magna* grazing activity. However, it is interesting to note that the inhibition limit for *D. magna* depends on their length. Thus, large *D. magna* produced higher filtration under higher dissipation rates than small *D. magna* individuals did. These results are in accordance with those found for the change in the allometric functions of *D. magna* velocity. Small scale turbulence has been also found to enhance development, excretion and feeding rates in other species such as copepods^50^, as well as increases in the heart beat rate of *D. pulex*^51^. Specifically, dissipation levels above 0.05 cm^2^ s^−3^ were found to increase the heart-beat rate by 14.3% for *D. pulex*. This critical dissipation found by Alcaraz *et al*.^51^, is in accordance to that found for the critical dissipation for the small *D. magna* of 0.04 cm^2^ s^−3^ in the present study, especially if we take into account that *D. pulex* have sizes below that of *D. magna*^45^.

The collision frequency function characterising the probability of encounter between a *D. magna* individual and the food particles themselves was calculated based on the model presented by Kiorboe and Saiz^8^. Equation (15) is used instead of β = GL^3^ ^7,34^ because the Kolmogorov length scale (λ) was lower than the *D. magna* length L^8^ (see Table 1). Our study provided capture efficiencies of 0.10, which are lower than those obtained by Kobayashi *et al*.^6^, who found capture efficiencies below 0.6 for collision of inorganic particles with different cohesiveness obtained by different salt concentrations. The low capture efficiency obtained in this study for *D. magna* might be due to the fact that not all their surface is efficient in capturing food and only the part of their body in charge of the uptake is responsible for the effective capture.

Zooplankton schooling can locally produce biogenic mixing in aquatic ecosystems, which results in turbulent kinetic energy dissipations to the order of 0.2 cm^2^ s^−3 ^^20^. Therefore, from the results obtained in the present study, flow dissipations above 0.16 cm^2^ s^−3^, close to the above mentioned 0.2 cm^2^ s^−3^ found for the produced dissipations of freely swimming *D. magna*^20^, have been found to negatively affect *D. magna* mobility, filtration and survival. Despite this, high dissipation rates have been found to markedly decrease *D. magna* survival after 24 h of exposure. Therefore, lakes and ponds with intermediate to large levels of turbulence might be especially dangerous environments for zooplankton due to enhanced kinetic mixing, which in most climate change scenarios is expected as a result of an increase in the frequency and intensity of storms. In high dissipation rate environments, the *D. magna* mortality increased by up to 80% after 24 hours of being submitted to strong dissipations. As a result, it is expected that at longer exposure times the capacity of filtering by the individuals will be compromised along with feeding strategies and feeding success. Although turbulence might enhance the food capture capacity of *D. magna* individuals^22,52,53^, at ε > ε_cr_, the mechanical movement was inhibited. Other authors have found turbulence to affect or disturb vertical refuge for zooplankton^54^ and/or zooplankton community composition through changes in phytoplankton^55^ and predation^56,57^. Therefore, high levels of turbulence will inhibit *D. magna* swimming, affect survival and increase the contact of *D. magna* individuals with their preys^4,8,58^. Meanwhile, low turbulence levels may enhance zooplankton development (gross growth and postembryonic development) through enhanced efficiency of food collection and ingestion^28^. This latter finding is also in accordance with other authors who have studied the clearance rate by zooplankton in sheared flows^26^.
